# Abscisic acid: Metabolism, transport, crosstalk with other plant growth regulators, and its role in heavy metal stress mitigation

**DOI:** 10.3389/fpls.2022.972856

**Published:** 2022-09-14

**Authors:** Sandeep Kumar, Sajad Hussain Shah, Yerramilli Vimala, Hanuman Singh Jatav, Parvaiz Ahmad, Yinglong Chen, Kadambot H. M. Siddique

**Affiliations:** ^1^Plant Physiology and Tissue Culture Laboratory, Department of Botany, Chaudhary Charan Singh University, Meerut, India; ^2^Soil Science and Agricultural Chemistry, Sri Karan Narendra Agriculture University Jobner, Jaipur, India; ^3^Department of Botany, GDC Pulwama, Jammu and Kashmir, India; ^4^The UWA Institute of Agriculture and School of Agriculture and Environment, The University of Western Australia, Perth, WA, Australia

**Keywords:** abscisic acid, heavy metal, growth, physiological processes, antioxidant, productivity

## Abstract

Heavy metal (HM) stress is threatening agricultural crops, ecological systems, and human health worldwide. HM toxicity adversely affects plant growth, physiological processes, and crop productivity by disturbing cellular ionic balance, metabolic balance, cell membrane integrity, and protein and enzyme activities. Plants under HM stress intrinsically develop mechanisms to counter the adversities of HM but not prevent them. However, the exogenous application of abscisic acid (ABA) is a strategy for boosting the tolerance capacity of plants against HM toxicity by improving osmolyte accumulation and antioxidant machinery. ABA is an essential plant growth regulator that modulates various plant growth and metabolic processes, including seed development and germination, vegetative growth, stomatal regulation, flowering, and leaf senescence under diverse environmental conditions. This review summarizes ABA biosynthesis, signaling, transport, and catabolism in plant tissues and the adverse effects of HM stress on crop plants. Moreover, we describe the role of ABA in mitigating HM stress and elucidating the interplay of ABA with other plant growth regulators.

## Introduction

Plants encounter a plethora of environmental stresses classified as biotic or abiotic stresses. Among abiotic stresses, heavy metal (HM) stress is a severe ecological restraint with detrimental effects on plant growth, metabolic processes, and crop productivity (Ghori et al., 2019). Agrarian soils in different parts of the world are slightly/moderately contaminated with HM, which is concerning due to their harmful effect on human health, ecosystem function, and global economic growth (Shahid et al., 2015). HM toxicity hampers normal plant functioning by producing excessive reactive oxygen species (ROS), repressing respiration, photosynthesis, and vascular and enzymatic activities, and disrupting ion homeostasis (He et al., 2021). Plants have complex inherent biological mechanisms to counter the adversities of HM but fail to mitigate them at toxic levels (Chandrakar et al., 2020). However, plant growth regulators (PGRs) play a crucial role in strengthening the tolerance mechanisms of plants against HM stress (Hasanuzzaman et al., 2018; Singh et al., 2020). PGRs are small chemical messengers that control various phenological characteristics, physio-biochemical, and developmental aspects in plants in a concentration of the molecular inducer-dependent and plant development stage-dependent manner (Vimala, 1985). The major PGR classes include abscisic acid (ABA), auxins, brassinosteroid (BR), cytokinins (CK), ethylene (ET), gibberellic acid (GA_3_), jasmonic acid (JA), salicylic acid (SA), nitric oxide (NO), and polyamines (PAs). The roles of PGRs in enhancing endurance against abiotic stresses, including HM, are well established (Saini et al., 2021; Shah et al., 2021, 2022; Kumar et al., 2022). This review focuses on the role of ABA in mitigating HM stress at the metabolic, transport, and crosstalk with other PGRs levels.

Abscisic acid is a small sesquiterpene molecule with many crucial functions in plant phenological and developmental processes, including seed dormancy and development, vegetative growth, flowering, stomatal movement, lipid and storage protein synthesis, and leaf senescence (Chen et al., 2020). Under HM stress, ABA modulates various physiological, biochemical, and molecular processes in plants (Vishwakarma et al., 2017). Exogenous ABA application can enhance plant tolerance to toxic metal stress. For example, 10 and 20 μM foliar ABA spray alleviated cadmium (Cd) inhibitory effects in *Lactuca sativa* L. by increasing its growth, chlorophyll content, photosynthetic efficiency, catalase activity, and proline and protein contents (Tang et al., 2020). Deng et al. (2021) reported that 5 μM ABA application mitigated the Cd-induced damage in roots of *Malus hupehensis* (Pamp.) by decreasing cell death and hydrogen peroxide and malondialdehyde contents. Further, 10 μM foliar ABA treatment ameliorated Cd toxicity in *Vigna radiata* L. by increasing chlorophyll content, osmolyte concentration, and antioxidant defense machinery (Leng et al., 2021).

It is well-accepted that ABA plays a vital role in improving morphological features, developmental aspects, and yield and quality parameters, and ameliorating the adversities of HM stress in crop plants. This review characterizes ABA biosynthesis, signaling, transport, and catabolism in plants and summarizes the impact of HM stress on crop growth, photosynthesis, and yield. Toxic metal and metalloids evolved with the evolution of green plants, accumulating in edible parts. Hence, plants evolved mechanisms to uptake, exclude, and mitigate the impact of unrequired or toxic metals. ABA is one such PGR evolved to mitigate HM stress.

## Abscisic acid biosynthesis

**FIGURE 1:**
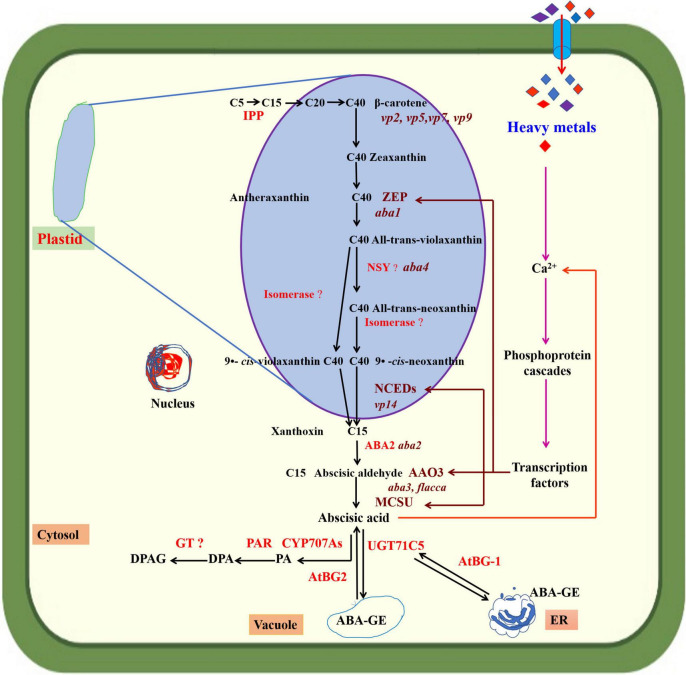
Abscisic acid (ABA) biosynthesis in plant cells. Endoplasmic reticulum (ER). The precursor of ABA in plants is (C40) β-carotene. Isopentenyl pyrophosphate (IPP) converts C5 to C15 and continues to C40. β-carotene converts C40 to xanthoxin (C15) in plastids and cytosol. Zeaxanthin epoxidase (ZEP), neoxanthin synthase (NYS), 9-*cis*-epoxycarotenoid dioxygenase (NCEDs), phasic acid (PA), dihydrophaseic acid (DPA), PA reductase (PAR), DPA-4-β-D-glucoside (DPAG), UDP-glucosyltransferase (UGT) encoded by (UGT71C5), cytochrome P450 monooxygenase (P450) encoded by (CYP707As), glycosyltransferase (GT), β-glucosidase encoded by (AtBG1 and AtBG2), ABA-glucose ester (ABA-GE), abscisic aldehyde oxidase (AAO3), and MCSU, molybdenum cofactor sulfurase.

Abscisic acid is a 15-carbon terpenoid molecule identified in the 1960s and recognized as a growth-inhibiting compound (abscisin II) associated with bud dormancy in maple and fruit abscission in cotton (Schwartz and Zeevaart, 2010). Later work revealed that ABA plays an important role in several developmental and physio-biochemical processes and regulates abiotic stress responses in plants (Sah et al., 2016). ABA is synthesized in all cells with chloroplasts or amyloplasts and found in major organs and tissues (Taiz et al., 2015). ABA biosynthesis starts in the chloroplast and ends in the cytoplasm (Seo and Koshiba, 2002). The isopentenyl diphosphate (IPP) is formed in plastids from pyruvic acid and glyceraldehyde 3-phosphate (Figure 1) and converted into geranylgeranyl diphosphate (GGPP) through the methylerythritol 4-phosphate (MEP) pathway. GGPP then forms phytoene (C_40_), which is converted into lycopene and β-carotene, and finally zeaxanthin. The conversion of zeaxanthin to all-*trans*-violaxanthin (C_40_) is mediated by the enzyme zeaxanthin epoxidase (ZEP). The all-*trans*-violaxanthin (C_40_) is then modified into 9-*cis*-neoxanthin (C_40_) and divided into xanthoxin (C_15_) and a C_25_ metabolite by 9-*cis*-epoxycarotenoid dioxygenase enzyme (NCED). This reaction is a rate-limiting step, and NCED is a crucial enzyme in ABA biosynthesis. Xanthoxin is transferred to the cytoplasm, where it is transformed into ABA-aldehyde by an enzyme that belongs to a short-chain dehydrogenase/reductase family. ABA-aldehyde is then converted into ABA with the reaction catalyzed by ABA-aldehyde oxidase (AAO) (Milborrow, 2001; Xiong and Zhu, 2003; Nambara and Marion-Poll, 2005; Mehrotra et al., 2014; Cardoso et al., 2020). Various enzymes, encoded by different genes, are involved in ABA biosynthesis. For instance, Arabidopsis aldehyde oxidases genes *(AAO3)* encode the AAO enzyme (Seo et al., 2000). The *ABA2* gene encodes the ZEP enzyme to enhance its activity. Increased upregulation of *ABA2* mRNA in genetically altered plants had a positive role in delaying germination and increasing ABA levels in mature seeds of transgenic plants (Frey et al., 1999). In *Arabidopsis thaliana* L., ABA biosynthesis is controlled by a small family of five *NCED* genes (*AtNCED2, 3, 5, 6*, and *9*) located in plastids. Moreover, there is a differential contribution of *NCEDs* to ABA synthesis during *Arabidopsis thaliana* L. growth and development; for instance, *NCED2* and *3* have high expression levels in roots and leaves but low expression levels for seed development, and *NCED5, 6* and 9 have increased expression levels during later maturation stages. Further, NCED expression is controlled by the transcription factor *MYB96*, with an essential role in seed dormancy (Tan et al., 2003; Lee et al., 2015; Ma et al., 2018). *ATAF1* (*Arabidopsis thaliana* activating factor 1) is a transcription factor that regulates *Arabidopsis thaliana* L. development and environmental stress responses. *ATAF1* promotes *NCED3* expression that controls ABA biosynthesis (Jensen et al., 2013). HM stress affects plant ABA biosynthesis and varies between species and the type and level of HM stress. HM affect ABA biosynthesis at lower concentrations but inhibit it at higher levels. Copper (Cu) suppressed the transcription of ABA biosynthetic genes (*StABA1* and *StNCED1*, *AAO3*) in *Solanum tuberosum* L., decreasing ABA content (Liu et al., 2020). Cu stress significantly increased ABA contents in roots, shoots, and leaves of *Helianthus annuus* L. seedlings (Zengin and Kirbag, 2007). Hou et al. (2010) reported that aluminum (Al) enhanced endogenous ABA production in roots and leaves of *Glycine max*. L. Exposure to cadmium (Cd) stress significantly increased leaf ABA concentration in *Phaseolus vulgaris* L. but inhibited ABA accumulation in dry excised leaves (Poschenrieder et al., 1989). Cd stress increased endogenous ABA levels in the roots of a non-hyperaccumulating ecotype of *Sedum alfredii* due to upregulated expression of ABA biosynthesis genes (*SaABA2*, *SaNCED*) (Tao et al., 2019). Further, Cd stress increased ABA content more in roots than old leaves of *Boehmeria nivea* L., with the lowest levels in new leaves. Long-time treatment of high Cd concentrations decreased endogenous ABA biosynthesis (Chen et al., 2021). HM compete with other metal ions and depolarize membrane-inducing Ca^2+^ efflux from organelles, initiating a Ca^2+^ signaling cascade of events that trigger the upregulation of ZEP, NCED, and MCSU genes (Ahmad et al., 2010).

## Abscisic acid transport

Abscisic acid is produced primarily in vascular tissue with its transportation among cells, tissues, and organs essential for regulating plant physio-biochemical processes under varying environmental responses (Kuromori et al., 2010; Saradadevi et al., 2016). ABA exists in anionic (ABA^−^) and protonated (ABAH) forms in plants. It is a weak acid, diffusing the bio-membrane passively when in protonated form (Ng et al., 2014; Chen et al., 2020). However, active ABA translocation across the plasma membrane can be mediated by transporters, including ATP-binding cassette (ABC) transporter proteins, nitrate or di/tri-peptide transporters [NRT1/PTR family proteins (NPF)], multidrug and toxic compound extrusion (MATE)-type/DTX transporters, and AWPM-19 family proteins (OsPM1). These transporters were first identified in *Oryza sativa* L. (Kanno et al., 2012; Zhang et al., 2014; Merilo et al., 2015). In eukaryotes, ABC transporters are categorized into sub-families from ABCA to ABCH. About 129 ABC transporters of ABA have been identified in *Arabidopsis thaliana* L. (Kang et al., 2010). Many ABCG members are suggested to play a role in ABA transportation. The AtABCG25 transporter, located in the plasma membrane of root vascular bundles and close to leaf vascular veins, exports ABA from vascular tissue to different plant parts (Kuromori et al., 2010; Andolfo et al., 2015). The AtABCG40 transporter mediates ABA import into plant cells, and its loss of function in guard cells delays stomatal closure by limiting ABA uptake (Kang et al., 2010). The AtABCG22 transporter may participate in stomatal regulation by promoting ABA uptake (Kuromori et al., 2011). AtABCG25 and AtABCG31 regulate ABA export from the endosperm to multiple sites in plants, whereas AtABCG30 and AtABCG40 import ABA into the embryo, controlling seed germination (Kang et al., 2015). AtDTX50 (DTX/MATE-type transporter) is localized in vascular tissue and guard cells, participating in ABA efflux in *Arabidopsis thaliana* L. and exogenous application of ABA upregulated *AtDTX50* gene expression (Zhang et al., 2014). Recently, Shimizu et al. (2021) detected two Arabidopsis NPF4.6 and NPF5.1 transporters in vascular tissues, guard cells, and leaf cell tissues that act as ABA importers to regulate stomatal movement by controlling ABA uptake. Studies have suggested that lower levels of HM contamination trigger ABA to increase inside plant cells, which is continuously transported throughout the plant, regulating plant defensive responses. However, under extreme HM stress, ABA synthesis and transport decreases due to blockages in the vascular transportation route. Under such conditions, exogenous supply of ABA can alleviate these perturbations.

## Abscisic acid catabolism

Catabolism and biosynthesis rates determine ABA hormone levels and are essential for modulating plant growth and metabolic processes in response to varying environmental cues (Kitahata et al., 2005). Endogenous ABA levels change with varying ecological conditions. Under favorable environmental conditions, ABA is metabolized into an inactive form through hydroxylation and conjugation pathways (Ng et al., 2014). In the hydroxylation pathway, ABA is hydroxylated by the oxidation of its three methyl groups in the ring structure (C-7′, C-8′, and C-9′). C-8′ is the major catabolic route, in which 8′-hydroxy-ABA is unstable and spontaneously converted into phaseic acid (PA) and dihydro-phaseic acid (DPA), catalyzed by cytochrome P450 type enzyme (CYP707A), an important enzyme in ABA metabolism (Saito et al., 2004; Baron et al., 2012; Rodriguez, 2016; Seo and Marion-Poll, 2019). *CYP707A* gene expression in guard cells and vascular tissue controls ABA metabolism and function under diverse conditions (Okamoto et al., 2009). During seed development and germination, members of the *CYP707A* gene family (*CYP707A1* to *CYP707A4*) help decrease endogenous ABA levels (Zheng et al., 2018). The inactivation of ABA is also mediated by the conjugation of ABA with glucose to form ABA glucose ester (ABA-GE) catalyzed by uridine diphosphate glycosyltransferases (UGTs). ABA-GE (inactive ABA product) is formed in the cytoplasm and stored in the vacuoles (Priest et al., 2006; Zhu et al., 2011; Chen et al., 2020). However, under abiotic stress, ABA-GE is converted back into free ABA through enzyme-catalyzed hydrolysis regulated by beta-glycosidase homolog 1 and 2 (BG1 and BG2) enzymes (Xu et al., 2012). Therefore, ABA catabolism significantly controls ABA levels under normal and adverse conditions. In summary, HM toxicity decreases endogenous ABA levels by inhibiting the expression of genes involved in ABA biosynthesis and thus halting the ABA-mediated physiological effect.

## Abscisic acid signaling

**FIGURE 2:**
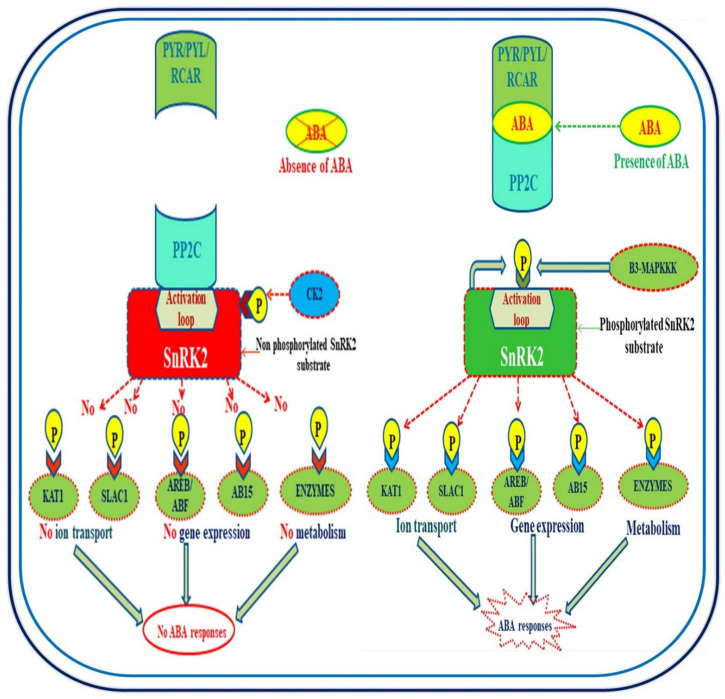
Schematic representation of the mechanism of action of abscisic acid (ABA). The core component of ABA signaling includes pyrabactin resistance 1 (PYR1)/PYR-Like (PYL), regulatory component of ABA receptors (RCARs), clade a protein phosphatases PP2C, ABA insensitive 1/2 ABI 1/2, hypersensitive to ABA1/2, HBA1/2, and sucrose non-fermenting-1-related protein kinase 2 family members (SnRK2s).

Understanding the mechanism of action of ABA is essential for plants to enhance their biological functions under unfavorable climatic conditions (Ali et al., 2020). Three main components are involved in ABA signaling: pyrabactin resistance (PYR)/pyrabactin resistance-like (PYL)/regulatory component of ABA receptor (RCAR), protein phosphatase 2C (PP2C; negative regulators), and sucrose non-fermenting (SNF1) related protein kinase 2 (SnRK2; positive regulators) (Ye et al., 2017). When ABA is present, PYR/PYL/RCAR-PP2C starts forming a complex structure that inhibits the negative effect of PP2C and further activators SnRK2 function for the phosphorylation of downstream proteins, including transcription factors to promote ABA-responsive gene expression (Yoshida et al., 2014). Figure 2 is a diagrammatic representation of the mechanism of action of ABA. In addition, HM contamination decreases endogenous ABA content and thus stopped ABA modulated processes due to the non-availability of ABA in cells.

## Impact of heavy metal stress on plants

Heavy metals are naturally occurring elements with high atomic weight and density, including some micro-nutrients crucial for plant growth and development. However, excess HM concentrations adversely affect plant growth and survival. The main HM-comprising metals and metalloids, such as cadmium (Cd), lead (Pb), arsenic (As), mercury (Hg), silver (Ag), aluminum (Al), and chromium (Cr), do not play vital roles in plant growth and development but their toxic levels disturb plant development and metabolism (Gill, 2014; Keyster et al., 2020). HM are released and accumulated in soils from natural sources and anthropogenic activities, including industrialization, smelting, extensive mining, fertilizer application, polluted water irrigation, fossil fuel combustion, and vehicle emitted gases and pollutants (Khan et al., 2017; Lajayer et al., 2019). Elevated HM levels increase ROS generation, causing metabolic imbalance, disturbing ion homeostasis, disorganizing the antioxidant defense system, disrupting protein structure, pigment synthesis, enzyme activities and membrane integrity, increasing lipid peroxidation, and thus decreasing plant growth and productivity (Emamverdian et al., 2015; Jalmi et al., 2018). HM stress adversely affects crop growth, physiological processes, and production (Table 1).

**TABLE 1:** Toxic effects of heavy metal (HM) stress on plant growth, physiology, and yield parameters.

Plant species	HM	HM dose	Impacts on plant	References
*Cicer arietinum* L.	Cd	23 mg kg^–1^ soil	Reduced plant biomass, nodule number, chlorophyll content, root and shoot N content, seed yield and grain yield	Wani et al. (2007)
*Zea mays* L.	Hg+Cr+Cd	151.9, 39.24, and 7.3 mg kg^–1^ soil	Decreased plant growth, N content, and seed protein content	Ghani (2010)
*Sorghum bicolor* L.	Cd	100 mg kg^–1^ soil	Minimized plant height and chlorophyll synthesis and increased lipid peroxidation	Da-lin et al. (2011)
*Glycine max* L.	Cd	100 mg kg^–1^ soil	Reduced photosynthetic efficiency, stomatal conductance, nitrate reductase activity and leghemoglobin content	Sunita et al. (2015)
*Eruca sativa* L.	Cd and Pb	200 mg and 1,500 mg kg^–1^ soil	Decreased stem diameter, leaf number and biomass and root fresh and dry weights	Yildirim et al. (2019)
*Morus alba* L.	Cd and Pb	100 and 200 μM L^–1^ nutrient solution	Decreased chlorophyll content, carbon fixation, rate and stomatal movement	Huihui et al. (2020)
*Sassafras tzumu* L.	Cd	100 mg kg^–1^ soil	Reduced plant height, biomass, chlorophyll content and photosynthetic rate	Zhao et al. (2021)
*Vigna radiata* L.	Cd	30 mg kg^–1^ soil	Decreased chlorophyll content, gas exchange parameters, soluble proteins, and free amino acids content	Aqeel et al. (2021)
*Citrus aurantinum* L.	Cu and Pb	800 μM (nutrient solution)	Decreased plant height, chlorophyll pigment, photosynthetic rate, antioxidant activity, mineral nutrient absorption and water balance	Giannakoula et al. (2021)

### Growth

Various studies have reported that HM contamination hinders crop plant growth features by altering root and shoot length morphology and anatomy and reducing plant biomass. For instance, 900 μM Cd and 1500 μM Pb reduced root and shoot lengths of *Brassica juncea* L. seedlings (John et al., 2009). In another *Brassica juncea* L. study, Cr, Pb, Cd, and Hg application at 100, 200, 10, and 50 mg kg^–1^ soil, respectively, decreased root and stem dry weights (Sheetal et al., 2016). Two *Zea mays* L. genotypes with contrasting root systems exhibited differential growth responses to moderate Cd stress (20 mg kg^–1^ soil) with less growth reduction in small-rooted genotypes than large-rooted genotypes (An et al., 2022a,b).

### Physio-biochemical processes

Heavy metal stress severely impacts physiological processes, including the electron transport system, enzymatic activities, nutrient uptake, photosynthetic pigments, and the photosystem. Moreover, high HM levels induce toxic ROS production, causing cellular damage by inactivating proteins and enzymes, increasing lipid peroxidation, damaging nucleic acids, and disrupting ion homeostasis and metabolic balance, leading to plant death (Goyal et al., 2020). In addition, HM decrease photosynthetic pigment content, negatively affect photosystems I and II, reduce transpiration rate, stomatal conductance, and water use efficiency, and disturb plant water relations and cellular nutrient balance (Małkowski et al., 2019). Studies have suggested that soil contaminated with HM alters overall plant physiology. For instance, a pre-sowing seed treatment with 100 μM Cd decreased the protein content in roots and peroxide activity in leaves of *Cicer arietinum* L. (Bavi et al., 2011). *Brassica juncea* L. grown in soil contaminated with Cr, Pb, Cd, and Hg at 100, 200, 10, and 50 mg kg^–1^ soil, respectively, had reduced photosynthetic rates and chlorophyll a and b contents (Sheetal et al., 2016). Soil-applied Pb (400, 800, and 1200 ppm) reduced photosynthetic pigments, antioxidant enzyme activities, increased hydrogen peroxide content, and lipid peroxidation and affected protein and proline content in *Oryza sativa* L. (Ashraf et al., 2017). *Tamarix usneoides* L. seedlings treated with 18 mg Cd kg^–1^ Cd had decreased chlorophyll contents and photosynthetic rates (Mayonde et al., 2021).

### Yield

Heavy metal toxicity induces deleterious effects on various physiological and biochemical processes, decreasing crop productivity (Shahid et al., 2015). Soil-applied As (30 mg kg^–1^) significantly decreased the number of panicles and filled grains per pot and total grain yield in *Oryza sativa* L. (Rahman et al., 2007). Soil incubated with 150 mg kg^–1^ Cr (VI) markedly decreased the yield of *Hordeum vulgare* L., but significantly increased the yield and ammonia nitrogen content of *Zea mays* L. (Wyszkowski and Radziemska, 2010). Arduini et al. (2014) reported that soil supplemented with 4.5 mg kg^–1^ Cd reduced plant height, biomass, and vegetative growth but increased dry matter and grain Cd accumulation in *Triticum durum*. Further, soil-applied Pb (400, 800, and 1,200 ppm) decreased tiller number per pot, grain number per panicle, 1000-grain weight, and grain yield in *Oryza sativa* L. Arduini et al. (2014). In another study, soil application of 100 mg kg^–1^ Cd significantly decreased pod number per plant, seed number per pod, seed weight, and seed yield in *Brassica juncea* L. (Irfan et al., 2015). Moderate Cd stress significantly reduced the grain yield of *Zea mays* L., especially under low nitrogen supply (An et al., 2022b). These studies suggest that HM stress impedes growth, physiological processes, and yield attributes, decreasing overall crop production.

## Role of abscisic acid in mitigating HM toxicity

**FIGURE 3:**
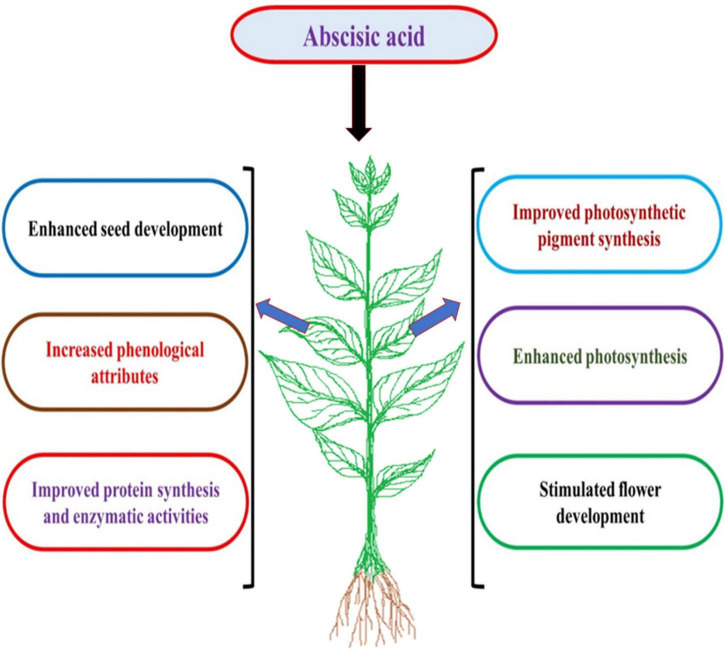
The effect of exogenous application of abscisic acid (ABA) on plant growth and physiological processes.

**FIGURE 4:**
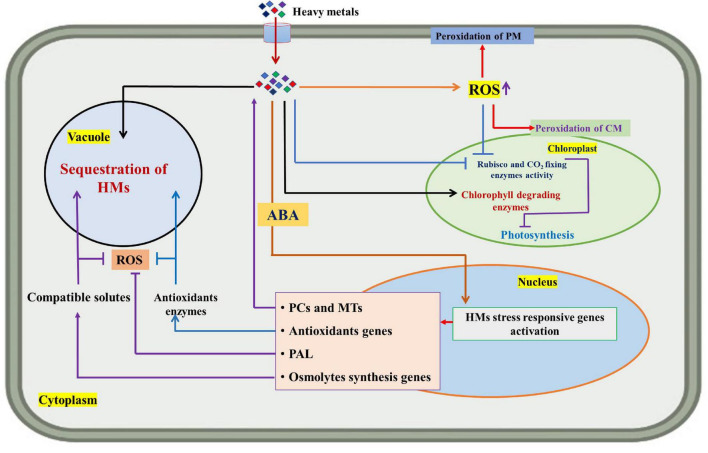
Diagram showing the sequestration mechanism of heavy metals (HMs) in plant cells and the role of abscisic acid (ABA) in mitigating HM stress. ROS (reactive oxygen species), PM (plasma membrane), CM (chloroplast membrane), PCs (phytochelatins), MTs (metallothioneins), and PAL (phenylalanine ammonia-lyase).

Abscisic acid is a prominent stress hormone that regulates numerous physiological processes and helps plants cope with the adverse impacts of HM stress (Shen et al., 2017). ABA regulates toxic metal translocation from roots to shoots. It closes stomata and reduces the transpiration rate, limiting the long-distance transfer of HM (Saradadevi et al., 2017; Hu et al., 2020). ABA enhances plant biomass, physiological processes, antioxidant defense system, and osmolyte accumulation, reducing the effects of HM stress. The activation of genes involved in ABA production increases endogenous ABA concentration in some plant species under Cd stress, including *Oryza sativa* L., *Solanum tuberosum* L., *Brassica napus* L., *Triticum aestivum* L., and others (Hsu and Kao, 2003; Stroiński et al., 2013; Shen et al., 2017; Hu et al., 2020). Exogenous ABA supply decreased transpiration rate and Cd concentration and increased Cd tolerance in *Oryza sativa* L. seedlings (Hsu and Kao, 2003). Further, exogenous ABA supplementation reduced the deleterious effects of Cd stress in *Brassica napus* L. by decreasing internal Cd accumulation and malondialdehyde content and enhancing fresh weight (Meng et al., 2009). According to Stroiński et al. (2013), *Solanum tuberosum* L. seedlings treated with 0.1 mM ABA increased StPCS1 content, the expression of genes coding 9-*cis*-epoxycarotenoid dioxygenase 1 and basic leucine zipper, phytochelatins synthase activity in roots, and the endogenous ABA level. In *Arabidopsis thaliana* L., Fan et al. (2014) and Pan et al. (2020) revealed that ABA reduced Cd absorption by inhibiting the process of iron-regulated transporter 1 (IRT1) and relieving Cd-induced toxicity. In *Populus euphratica* L. cells, ABA alleviated 100 μM Cd stress by improving antioxidant enzyme activity that scavenged H_2_O_2_ to prevent Cd from entering H_2_O_2_-induced Ca^2+^-permeable channels (Han et al., 2016). ABA application at 20 μM L^–1^ improved Cd extraction and phytoremediation activity in *Solanum photeinocarpum* L. (Wang et al., 2016). The *JrVHAG1*gene functions as a Cd stress response regulator in the ABA signaling pathway and transcription regulation network of *MYB* in plants (Xu et al., 2018). *Vitis vinifera* L. seedlings treated with 10 μM ABA reduced Zn stress by decreasing Zn uptake and accumulation in roots and promoting the expression of the *ZIP* gene and detoxification-related genes in leaves and roots (Song et al., 2019). ABA-treated *Populus alba* L. seedlings had less Pb toxicity and increased expression of some genes involved in Pb transport and detoxification, including *NRAMP1.4, ABCG40, PCS1.1, FRD3.1*, and *ABCC1.1* (Shi et al., 2019). In *Lactuca sativa* L. seedlings, exogenously applied ABA decreased Cd absorption and relieved Cd toxicity (Dawuda et al., 2020). In *Sedum alfredii* L. seedlings, exogenously applied ABA (0.2 mg L^–1^) alleviated Cd stress by increasing endogenous ABA content through enhanced ABA synthetase activity. It also promoted the expression of *HsfA4c* in roots and *NAS* and *HMA4* in shoots to improve Cd resistance (Lu et al., 2020). Exogenous ABA application minimized the damage of excess Zn by increasing endogenous ABA concentration, proline accumulation, and ascorbate peroxidase (APX) activity and inhibiting lipoxygenase (LOX) activity (Li and Song, 2020). ABA could also be involved in upregulating the *APX* gene and proline synthesis genes and downregulating the *LOX* gene in Arabidopsis thaliana L. (Li and Song, 2020). Exogenous ABA application at 40 and 60 μM L^–1^ ameliorated Cd and Pb toxic effects by improving total chlorophyll content and antioxidant capacity in Sedum alfredii L. and *Hylotelephium spectabile* L. (Cheng et al., 2022). Seed priming with ABA also reduced HM toxicity in plants by increasing glutathione content, non-protein thiol and cysteine, and phytochelatins synthesis (Saha et al., 2021). ABA reduced Zn stress damage by enhancing proline content and antioxidant enzyme activities in *Robinia pseudoacacia* L. (Lou et al., 2021). ABA also regulated Cd hyperaccumulation in Sedum alfredii by lowering aquaporin expression in roots, the number of xylem vessels in stems, and stomatal size and density in leaves (Tao et al., 2021). Chen et al. (2022) suggested that exogenously applied ABA improved the phytoremediation efficiency of Sedum alfredii grown in Cd, Pb, and Zn contaminated soil. Figure 3 illustrates the role of exogenously applied ABA in plants. Figure 4 and Table 2 overview HM sequestration in plant cells mediated by ABA.

**TABLE 2:** Alleviation of heavy metal (HM) toxicity in plants by abscisic acid (ABA).

Plant species	Type of HM stress and concentration	Growth conditions	ABA dose	Plant response	References
*Brassica napus* L.	Cd (100 μM)	Hydroponic	10 μM	Enhanced plant biomass and decreased Cd accumulation and malondialdehyde content	Meng et al. (2009)
*Populus euphratica* L.	Cd (100 μM)	Culture medium	5 μM	Improved cell survival and proliferation and antioxidant enzyme activities	Han et al. (2016)
*Brassica campestris* L.	Cd (100 μM L^–1^)	Hydroponic	5 μM	Increased soluble protein and chlorophyll content, antioxidant defense system, and Cd in plant roots	Shen et al. (2017)
*Populus alba* L.	Pb (3 mM)	Soil	10 μM	Improved root biomass, photosynthetic rate and ascorbate and glutathione contents	Shi et al. (2019)
*Lactuca sativa* L.	Cd (10 μM L^–1^)	Soil	5 μM	Enhanced plant biomass, stomatal conductance, internal CO_2_ concentration, antioxidant enzyme activity, proline content and soluble protein content.	Tang et al. (2020)
*Arabidopsis thaliana* L.	Zn (200 mg L^–1^)	Culture medium	15 μM	Increased proline accumulation, antioxidant enzyme activities, and endogenous ABA level	Li and Song (2020)
*Vigna radiata* L.	Cd (100 μM)	Seed tray	10 μM	Affected plant tolerance by stimulating antioxidant enzyme activity and inhibiting lipid peroxidation	Leng et al. (2021)
*Oryza sativa* L.	As (50 μM)	Culture medium	10 μM	Increased osmolyte concentrations, glutathione content, non-protein thiol, phytochelatins and glutathione reductase activity	Saha et al. (2021)
*Solanum lycopersicum* L.	Co (400 μM L^–1^)	Hydroponic	10 μM	Improved proline content and antioxidant enzyme activities, reduced Co translocation from roots to shoots	Kamran et al. (2021)
*Perilla frutescens* L.	Cd (10 mg kg^–1^)	Soil	5 μM	Enhanced plant biomass, photosynthetic pigments and antioxidant activities.	Shu et al. (2021)

## Crosstalk of abscisic acid with other plant growth regulators

Abscisic acid and auxin regulate several facets of plant growth and metabolism, mainly in a contrary manner (Sun and Li, 2014). For example, for seed germination and root growth, ABA-induced expression of Auxin Response Factor 2 (ARF2) and *arf2* mutants enhanced ABA sensitivity in Arabidopsis thaliana L. (Wang et al., 2011). ABA reduced cell division and altered auxin distribution more in *arf2* mutants than wild species. Exogenous ABA application to the *arf2* mutant reduced root length (Wang et al., 2011, 2013). ABA can also restrict primary root development by controlling cell proliferation in root tips (Wang et al., 2011). ABA slows seedling growth by increasing auxin signaling (Rock and Sun, 2005). The gene expression network containing the transcription factor ABA insensitive three regulates seed dormancy mediated by ABA and auxin *(ABI3).* Auxin controls *ABI3* gene expression during seed germination by selecting auxin response factors 10 and 16 (Wang et al., 2011; Liu et al., 2013). Auxin is required for seed dormancy while ABA suppresses seed germination, indicating that auxin and ABA have an interacting role in seed dormancy. Auxin acts in two directions in ABA modulated processes by (1) activating the ABA response and (2) improving biological ABA synthesis. However, endogenous and exogenous indoleacetic acid (IAA) inhibited seed germination in radical protrusions experiments, showing that ABA and auxin work together to prevent seed germination (Liu et al., 2013; Matilla, 2020). In the absence of ABA, an equal amount of IAA did not limit seed germination, suggesting that inhibition of seed germination mediated through auxin depends on ABA. In addition, basipetal transport of auxin increased with osmotically induced ABA by increasing AUX1 (Auxin Influx Transporter) and PIN2 (Auxin Efflux Transporter) (Xu et al., 2013; Wu et al., 2020). As a result, auxin and ABA communicate *via* the auxin response pathway to control plant growth and development.

Many aspects of plant development, such as seed dormancy and maturation, main root growth, and flowering are controlled by the antagonistic interactions of ABA and gibberellins (GA). ABA inhibits seed germination by promoting seed dormancy, whereas GA stimulates seed germination and promotes seedling growth (Yuan et al., 2011). Sustaining germination and dormancy requires balancing ABA and GA production and catabolism (White et al., 2000; Bicalho et al., 2015). In *Arabidopsis thaliana* L., exogenous ABA supply decreased primary root development, whereas GA promoted root growth (Ubeda-Tomás et al., 2008; Zhang et al., 2010; Shu et al., 2018). Similarly, ABA and brassinosteroids (BR) coordinate antagonistically to modulate plant physiological functions. It is well-known that ABA inhibits seed germination while BR promotes seed germination (Saini et al., 2015). Further, ABA has a regulatory effect on BR signaling. ABA components like AB11 and ABA12 interplay with BR signaling components such as BIN2 and BR11 receptors (Yang et al., 2016). The interaction of ABA and GA3 hormones with Pb and Zn may increase total phenolic content; however, their interaction with Cd prevents an increase in phenolic compounds (Ahmad et al., 2015). ABA is also involved in the interaction between BR and CKs, as it suppresses BR synthesis during metal toxicity (Zhang S. et al., 2009). However, the molecular interactions between ABA and GA and ABA and BR for regulating plant physiological processes need further investigation.

Abscisic acid and cytokinin interact to regulate the stress response. The enzyme cytokinin oxidase encoded by the multigenic family cytokinin oxidase (*CKX*) has several members responsible for cytokinin breakdown (Avalbaev et al., 2012; Sirko et al., 2021). For instance, ABA decreased the expression of *CKX* genes in *Arabidopsis thaliana* L. Under stress conditions, cytokinin receptor kinases (AHK2 and AHK3) negatively controlled ABA and the expression of genes responsible for osmotic stress, while mutants with cytokinin deficiency had increased survival rates (Werner et al., 2006; Tran et al., 2007).

Abscisic acid and ethylene work together to control stomatal closure, with ABA controlling the increase in ethylene concentration in leaves (Chen et al., 2013), while ethylene blocks the ABA signaling pathway and leads to slow stomatal closure (Harrison, 2012; Arc et al., 2013). ABA inhibits the expression of the ethylene biosynthesis gene (*ACS5*) that encodes an enzyme for determining the rate-limiting step of ethylene biosynthesis. ABA also positively controls ethylene response factor 11 (ERF11) behavior through the long hypocotyl5 (HY5) replication factor, an important molecular contact between ABA and ethylene synthesis that represses *ACS5* gene expression. The regulation of *YF-AtERF11* expression is crucial for ABA-controlled ethylene biosynthesis (Li et al., 2011; Corbineau et al., 2014). Exogenous SA mitigated Cd stress in *Triticum aestivum* L. by increasing GSH content, resulting in metal detoxification and scavenging ROS induced by HM-triggered ethylene production. SA supplementation increased ABA levels in wheat seedlings under Cd stress, which was attributed to *de novo* ABA biosynthesis. Further, endogenous ABA-controlled SA-mediated changes in the concentration of dehydrin proteins under HM stress demonstrated the protective mechanism of SA in *Triticum aestivum* L. (Kovács et al., 2014; Shakirova et al., 2016).

A complex interaction between ABA and the jasmonate (JA) signaling pathway controls plant protection and gene expression. Exogenous ABA application reduces JA-induced transcription of defense genes. In contrast, mutations in the ABA synthesis genes *aba1* and *aba2* caused the overexpression of JA-responsive genes (Anderson et al., 2004). Several components of ABA and the JA-ethylene signaling pathway interact antagonistically to control the expression of several stress-responsive genes in response to abiotic and biotic stressors (Anderson et al., 2004; Proietti et al., 2018). Most JA signaling pathway components in *Arabidopsis thaliana* L. are regulated by the transcription factor MYC2. Furthermore, overexpression of the *MYC2* gene in genetically engineered plants has been linked to increased ABA sensitivity and activation of numerous ABA-induced genes. In contrast, *Myc2* mutants with *Ds* mutations are less susceptible to ABA and have lower gene expression (Chen et al., 2011). As a result, MYC2 in *Arabidopsis thaliana* L. is an integrative hub for controlling JA and ABA signals (Sun and Li, 2014).

Abscisic acid and nitric oxide (NO) may interact to regulate plant growth and physiological processes, including seed germination, root growth, stomatal movement, and antioxidant enzyme activities (Hancock et al., 2011; Prakash et al., 2019). Increasing ABA levels in tobacco by increasing the expression of the 9-*cis*-epoxycarotenoid dioxygenase gene (*SgNCED1*) increased H_2_O_2_ and NO levels in mesophyll and guard cells, increasing the replication and activity of many antioxidant enzymes (Zhang Y. et al., 2009). The breakdown of seed dormancy in *Arabidopsis thaliana* L. caused rapid ABA depletion due to rapid NO accumulation, with the opposite effect during seed germination (Liu et al., 2009; Signorelli and Considine, 2018). Moreover, in guard cells, ABA-induced endogenous H_2_O_2_ production increased NO production to control stomatal movement (Bright et al., 2006; Saradadevi et al., 2014). Furthermore, exogenous NO application alleviated the inhibitory effect of ABA on leaf senescence in *Oryza sativa* L. (Hung and Kao, 2003). ABA and NO modulate metabolic plant functions in response to environmental stress by improving osmolyte synthesis and the antioxidant defense system (Iqbal et al., 2021, 2022). During HM stress, NO may interact with ABA to weaken HM stress. NO reduced the As-induced upregulation of ABA content in *Vicia faba* L., reducing As toxicity (Mohamed et al., 2016). Sadeghipour (2017) revealed that exogenous application of NO to *Vigna unguiculata* L. reduced ABA content under Pb stress, enhancing Pb tolerance. Wu et al. (2018) reported that molybdenum (Mo) induced ABA synthesis in *Triticum aestivum* L. by upregulating aldehyde oxidase activity. The NO acting downstream of ABA was involved in oxidative stress induced by Mo, implying an intermediate linkage in the relationship between ABA and NO.

As a result, ABA is a crucial plant growth regulator that coordinates with other phytohormones to control plant growth and development under adverse environmental conditions. Further research is needed on the molecular mechanisms behind complex signaling networks of ABA and other hormones.

## Conclusion

Heavy metal toxicity is a severe environmental restraint that hampers plant growth and productivity of food crops. PGRs are small signaling molecules that regulate many physiological and molecular processes in plants. Exogenous application of PGRs is an efficient approach to ameliorating the deleterious effects of HM and improving plant morphological characteristics, physio-biochemical processes, and production. As a vital PGR, ABA controls many developmental and metabolic functions of plants. This review revealed that exogenous ABA application alleviates the toxic effects of HM in plants by improving osmolyte synthesis, antioxidant machinery, phytochelatins synthesis, and enzymatic activities. It is also well-known that ABA has a significant role in providing abiotic stress tolerance in plants and improving plant growth and production. ABA application through the soil, foliar spray, seed priming, and culture media positively enhances crop productivity and thus could be used to improve the growth and yield of economically important crops. ABA plays a crucial role in mitigating HM stress by strengthening the tolerance mechanism of plants. The available literature on the role of ABA in HM stress mitigation offers a brief insight into morphological and physio-biochemical functioning and modulation in plants under HM stress. Thus, more research is needed to explore the molecular mechanisms of HM sequestration regulated through ABA to (1) enhance our understanding of ABA homeostasis during the plant life cycle, (2) identify crosstalk mechanisms between ABA and other PGRs under HM and other abiotic stresses and (3) explore the dynamic role of ABA in crop plants using novel genomic, molecular, and proteomic approaches.

## Author contributions

SK and SHS wrote and drafted the review article. YV, HSJ, PA, YC, and KHMS revised and edited the manuscript. All authors contributed to the article and approved the submitted version.
